# A Machine Learning Application to Camera‐Traps: Robust Species Interactions Datasets for Analysis of Mutualistic Networks

**DOI:** 10.1002/ece3.72584

**Published:** 2026-01-21

**Authors:** Pablo Villalva, Pedro Jordano

**Affiliations:** ^1^ Center for Sustainable Landscapes Under Global Change, Department of Biology Aarhus University Aarhus Denmark; ^2^ Integrative Ecology Group. Estación Biológica de Doñana, EBD‐CSIC Sevilla Spain; ^3^ Department of Biología Vegetal y Ecología Universidad de Sevilla Sevilla Spain

## Abstract

Recording and quantifying ecological interactions is vital for understanding biodiversity, ecosystem stability, and resilience. Camera traps have become a key tool for documenting plant–animal interactions, especially when combined with computer vision (CV) technology to handle large datasets. However, creating comprehensive ecological interaction databases remains challenging due to labor‐intensive processes and a lack of standardization. While CV aids in data processing, it has limitations, including information loss, which can impact subsequent analyses. This study presents a detailed methodology to streamline the creation of robust ecological interaction databases using CV‐enhanced tools. It highlights potential pitfalls in applying CV models across different contexts, particularly for specific plant and animal species. The approach aligns with existing camera trap standards and incorporates complex network analysis tools. It also addresses a gap in ecological research by extending the methodology to behavioral studies using video‐based image recognition, as most current studies rely on still images. The study evaluates CV's performance in estimating species interaction frequency (PIE) and its ecological implications. Results show that up to 10% of pairwise interactions may be missed with CV, with information loss varying among focal species and individual plants. The loss of information is minimal compared to the vast data CV enables researchers to gather especially if data is intended to be used in community‐level approaches where only three out of 344 unique pairwise interactions were missed. In community‐level approaches, the overall estimates of both PIEs and interaction strengths remained largely unaffected. The methodology provides a valuable resource for ecologists seeking to document ecological interactions efficiently. It offers guidelines for collecting reliable data while addressing CV's limitations in capturing unbiased species interaction data. Despite its constraints, CV significantly enhances the ability to gather large‐scale interaction data, particularly at the community level, making it an indispensable tool for ecological research.

## Graphical abstract

Our study includes novel components, focusing on plant‐animal interaction data as study case. We provide comprehensive guidelines and a proven methodology, aligned with existing camera trap standards, for the creation of reliable datasets, as well as a detailed discussion of limitations and logistic challenges. Beyond presenting AI performance metrics, we offer a complete overview of the ecological implications and rationale behind applying AI when generating species interaction data, particularly relevant for ecological networks analysis.

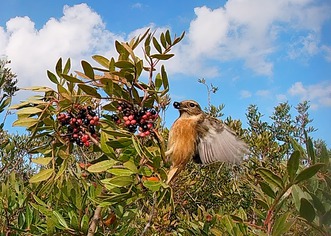

## 1 Introduction

No species on Earth lives without interacting with other species, the reason why ecological interactions are at the core of the Web of Life (Thompson 2009). These interactions play a vital role in supporting Earth's systems and are crucial for understanding ecosystem functioning (Loreau et al. 2001), leading to an increasing consideration as part of biodiversity (Jordano 2016a; Pollock et al. 2020). However, monitoring ecological interactions presents a significant challenge due to their labor‐intensive nature, often resulting in incomplete data samples, a problem analogous to any biodiversity sampling (Gotelli and Colwell 2011; Jordano 2016b).

**FIGURE 1:**
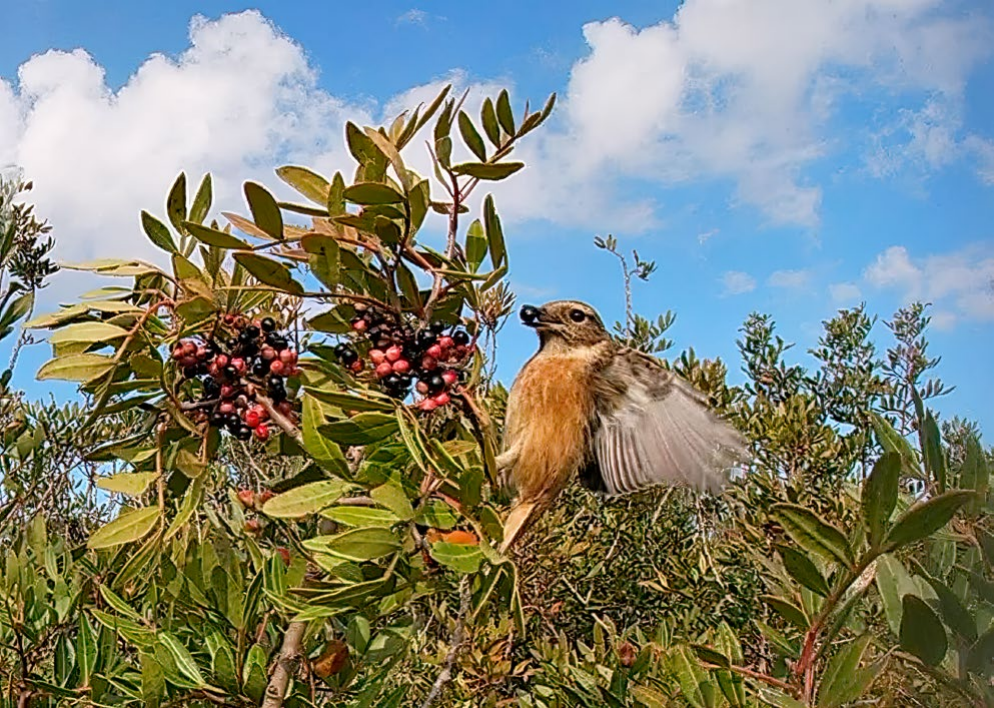
Organism photograph. Image from camera‐trap recording a female Stonechat ( *Saxicola rubicola* ) visiting a plant of *Pistacia lentiscus* . Stonechats typically pick the fruits on the wing, flycatching from a nearby perch to the infructescence. Camera‐traps allow not just the recording of interaction occurrence; also, detailed analysis of feeding behavior, fruit handling, fruit ingestion rate, fruit handling failures, etc., can be obtained when set in video mode. Location: Doñana National Park, Huelva, Spain. See Quintero et al. (2024).

Recent use of camera traps for wildlife monitoring has significantly advanced our understanding of vertebrate ecology and population structure (O'Connell et al. 2011). It is a cost‐effective method for monitoring multiple species over large spatial and temporal scales; however, the time required to process the data can limit the efficiency of broad‐scale surveys. Remote cameras have been used in studies ranging from population (Gardner et al. 2010) and community‐scale (Ahumada et al. 2011) to species distributions (Rich et al. 2017; Tobler et al. 2015). It is a non‐invasive method with wide application in behavioral studies (Caravaggi et al. 2020) as it offers information that allows us to record natural behaviors of animals without human disturbance in natural environments, especially when used in video mode (Figure 1). Recent studies have focused on diverse behavioral aspects, including daily activity patterns (Frey et al. 2017), road crossing behavior (Villalva et al. 2013), human‐wildlife conflicts (Johnson et al. 2006), and scent marking behavior (Rafiq et al. 2020). However, complex ecological processes such as species interactions have only been inferred using camera traps (e.g., Selwyn et al. 2020), and the application of camera traps in behavioral studies, particularly regarding foraging behavior (e.g., Koike et al. 2012) is still in its infancy.

Effective pipelines for handling large volumes of videos currently lack standardization and are underdeveloped for ecological purposes like monitoring ecological interactions. Recent advancements in Artificial Intelligence (AI) applied to image recognition—hereafter Computer Vision (CV) – (Leorna and Brinkman 2022; Vélez et al. 2023; Petroni et al. 2024), along with the decreasing cost of camera trap devices, allow the collection and management of high‐quality data within a reasonable timeframe and with an affordable budget. CV enables the processing of millions of images in a short time; yet, substantial and diverse pre‐processed data is necessary, including pre‐processing of images, model training, classification, manual quality checks, and data formatting (Böhner et al. 2023; Celis et al. 2024). Although CV has demonstrated accurate species identification (Gómez Villa et al. 2017; Whytock et al. 2021) and counts (Norouzzadeh et al. 2018), current models have primarily been trained for mammals due to the greater availability of information for them (Burton et al. 2015). Furthermore, the performance of CV approaches might be compromised when models are developed using training datasets that are unbalanced (Gómez Villa et al. 2017) or small and geographically limited (Tabak et al. 2019), or when applied to low‐resolution images (Gómez et al. 2016). Additionally, the process of model creation and refinement demands technical and programming expertise that extends beyond the capabilities of many field ecologists (Christin et al. 2019; Tabak et al. 2020). Nevertheless, current CV models can accurately classify images to remove blank (empty) ones (Beery et al. 2019) making it possible to select for detailed review just those images containing animals, notably reducing the revision effort while minimizing the tagging error by low‐trained models. While CV methods applied to camera traps have advanced rapidly in recent years, the vast majority have focused on still image analysis. Applications for processing and analyzing camera trap video sequences have received relatively little attention compared to static image‐based approaches. We leveraged current image recognition techniques to eliminate blank images, with direct application to the estimation of species' interaction frequencies in behavioral analysis based on video footage (Villalva et al. 2024).

This study presents a workflow for efficiently managing large volumes of video data to analyze plant–animal interactions, using automated image recognition specifically for camera trap surveys using videos, and introducing standardized procedures for analyses of plant–animal interactions. The workflow aimed to reduce time and effort in data compilation, supporting multi‐species monitoring across diverse ecosystems. It combines a field protocol for camera trap operations and data standards with advanced CV and a viewer program to streamline image review and tagging. Additionally, it incorporates methods to automate sampling effort data acquisition and infer interactions from short video sequences, minimizing labor‐intensive tasks. The ultimate goal is to create an accurate, fully annotated dataset documenting plant–animal interactions while optimizing efficiency.

## 2 Methods

Our approach for monitoring plant–animal interactions in natural habitats involves the strategic placement of camera traps, aimed at specific plant species, referred to as focal plants, to record interactions with animal frugivores that may mediate in seed dispersal. Not all plant species are suitable for monitoring with camera traps due to differences in growth habit, fruiting display, or site conditions that limit effective camera placement. In such cases, alternative methods may be more appropriate for documenting plant–animal interactions (Quintero et al. 2022). For this study, we monitored a total of 10 of the 28 plant species bearing fleshy fruits in Doñana National Park, including 119 individual plants. The plant communities studied include: (i) *Juniperus*‐dominated woodlands, (ii) Sclerophyllous scrublands primarily dominated by *Pistacia lentiscus* , alongside other fleshy‐fruited species, (iii) Scrublands comprising a variety of fleshy‐fruited plant species, occasionally dominated by *Arbutus unedo* , *Olea europaea var. sylvestris*, or *Myrtus communis* , (iv) Humid scrublands located in depressions (“monte negro”), predominantly dominated by *Rubus ulmifolius* , and (v) Coastal dunes characterized by dominant species such as *Corema album* or *Juniperus oxycedrus subsp. macrocarpa*. We use replicated individual plants of the same species and deploy cameras in each one (see Supporting Information S2 in Data S1 for details and specific recommendations). This monitoring approach allows data to be either pooled to represent species‐level interaction patterns for *community‐based* approaches or analyzed separately to explore variation among individual plants for *individual‐based* approaches. The goal is to obtain accurate records of visits to the plants by animals, allowing estimates of interaction probability (*PIE*, probability of interspecific encounter; Poisot et al. 2016). *PIE*_*ij*_ is thus the probability that plant species *i* establishes an interaction with animal species *j*, or, at the level of individual plants, the probability that an individual plant, *i*, interacts with animal frugivore species *j*. We deployed a total of 60 cameras, including 50 Browning Dark Ops and 10 Bushnell Trophy cam Aggressor. The cameras were strategically positioned towards different plant individuals bearing fleshy fruits and triggered by motion. Regular checks of the camera traps (batteries and SD‐cards restarting) were conducted at regular intervals, either weekly or biweekly, depending on the plant species and the specific period of the year. Cameras were set in video mode (10 s videos), an added value to provide valuable insights for species identification and especially their behavior, as well as an accurate quantification of fruit consumption and fruit handling, which become possible in some of the sequences obtained (not included in the analysis of this paper).

Interaction frequencies, *PIEs*, etc., can be derived by tallying those video sequences with evidence of an animal frugivore present while foraging at the focal plant. Active foraging for fruits by the animal can be evidenced by the video sequence showing an actual event of fruit picking, handling, and, maybe, fruit ingestion or fruit dropping. The interaction can also be inferred when the animal is visualized on the plant when actively foraging (despite no evidence of fruit feeding); and in order not to overestimate interaction frequency, such foraging events can be weighted by the actual proportion of fruit food in the diet of the animal species recorded (Quintero et al. 2024). Due to the typically short recording times of the cameras when triggered for memory saving (usually 10–20 s sequences), interaction frequencies can be drastically underestimated if just those sequences with actual fruit handling/ingestion are considered: a high frequency of false negatives (FN) would be discarded, when the short, recorded sequence is actually part of a feeding sequence at the plant including fruit ingestion. It is extremely rare that video sequences of 10–20 s span the total length of a frugivore's visit to a fruiting plant and include the actual fruit/seed handling or ingestion. Thus, including records with the presence of the animal at the plant, close to infructescences with ripe fruits, and showing active foraging behavior of fruit seeking helps minimize such underestimation bias due to FN (see below, *Stage‐2. Processing*).

In addition to the identification of animal species and their behavior, for some video recordings, we can accurately measure fruit consumption and determine fruit feeding behavior and consumption rates (see, e.g., Snow and Snow 1988; Moermond and Denslow 1985 for a full description of such diversity of fruit feeding behaviors).

### 2.1 Image Recognition Model

With camera‐trap monitoring under windy conditions, the movement of grasses and tree branches can easily lead to incorrect triggering, resulting in a significant number of empty images. This issue becomes particularly relevant in plant–animal interaction surveys focused on frugivory and pollination, where the natural environment must be unaltered and removal by hand of grass or branches is not an option. Thus, empty images may constitute a substantial portion of the video collection. To address this challenge, several image recognition models have been developed to eliminate empty images (Chalmers et al. 2023; Ahumada et al. 2020; Tabak et al. 2022). We used MegaDetector (MD), an open‐source model based on a deep convolutional neural network architecture designed for object detection (Beery et al. 2018), which is trained on millions of camera trap images from a wide range of ecosystems worldwide. The model detects and classifies images containing humans, animals, and vehicles, returning confidence scores for each detection. We applied a confidence threshold of 0.8 to minimize false positives (FP) and maximize the likelihood of true detections. Images with no detections above this threshold were classified as blank. While MegaDetector has shown high accuracy, some limitations remain, particularly for detecting small or partially obscured animals, so a manual verification step was performed on a stratified random subset of images to evaluate model performance and correct any systematic misclassifications (see below).

### 2.2 Protocol Pipeline

**FIGURE 2:**
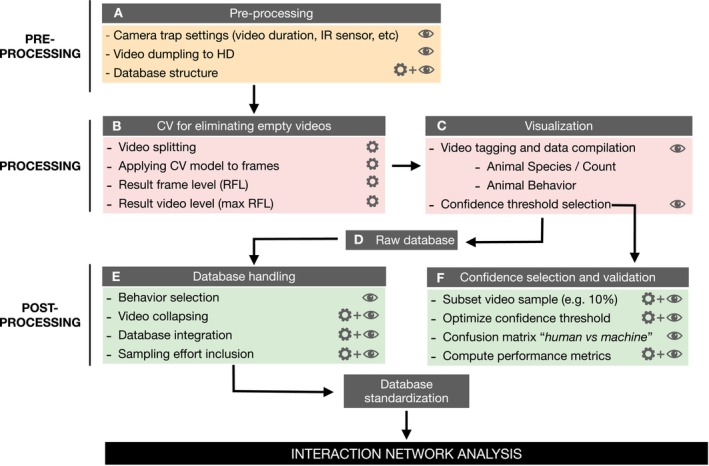
Detailed workflow for optimizing data obtained during sampling of interaction events between animal frugivores and fleshy‐fruit producing plant species using camera traps deployed in the vicinity of fruiting plants. The raw data obtained represent the interaction frequency between each animal species and the plant species visited, estimated from the number of 10 s video sequences obtained during camera operation whenever the camera is activated by movement by the frugivore species. The protocol includes steps for data pre‐processing, splitting of sequences to avoid pseudo‐replicated records, proper identification of sequences containing animal activity (i.e., the camera was actually triggered by animal activity and not by wind, etc.), and sequence analysis and compilation of distinct interaction records. Icons indicate whether a step is fully manual (eye symbol), fully automated (gear symbol), or involves human oversight and decision of an automated process (gear and eye combined).

Our streamlined workflow comprises three stages (Figure 2): (1) pre‐processing, (2) video processing, and (3) post‐processing. During stage 1, we establish a standardized data structure and procedure for camera trap settings, file archiving, and sampling effort automatization (Figure 2A, Table S.2.1 in Data S1). This ensures the consistency and accuracy of data collection, avoiding early errors that would cascade down the process chain. In stage 2, we leverage image recognition (CV) to classify and select just the videos featuring animals for further visualization (Figure 2B). Additionally, we use time‐saving software to visualize and tag the selected videos, properly identifying the interaction events and associating metadata to foraging behavior, etc., if available (Figure 2C). Finally, in stage 3, we integrate datasets from different video batches, encompassing various seasons, focal species, or camera sources (Figure 2E).

#### 2.2.1 Stage‐1. Pre‐Processing

The deployment of multiple cameras in replicated positions requires regular checks, resulting in a large number of videos with identical names and dates. Thus, an effective organization and management of such a vast and intricate dataset for a successful database creation is essential. We adapted the CamTrap Data Package structure (Bubnicki et al. 2024) and developed a standardized framework for controlling camera‐trap data for ecological interactions data gathering (Figure 2A). This ensures consistency and minimizes the risk of errors. It comprises three related plain texts containing all essential information for convenient data management and analysis (Table 1, Tables S.1.1–S.1.3 in Data S1).

**TABLE 1:** The main data is structured in three related plain text files (.csv).

File	Description
deployment.csv	Table with camera trap deployments.
video.csv	Table with media files captured by camera traps.
observation.csv	Table with observations based on media files (after visualization).

*Note:* Table *deployment* includes delpymentID, Location, and camera Setup information for each camera. Table *video* contains information related to each field revision including dates and TimestampIssues. Table *observation* contains video‐level information obtained from visualization.

Camera trap settings for data gathering in our case study with frugivory interactions involve several key aspects (Table S.2.1 in Data S1). First, it is advisable to use a consistent camera trap model throughout the study to maintain data integrity and reduce bias since detection may vary among different camera models and makes (Palencia et al. 2021). If using multiple camera models is unavoidable, a systematic rotation of deployments could be considered, although this may complicate keeping track of camera identification throughout the workflow. The sensitivity of the Infrared Sensor should be set carefully, considering both the surrounding environment and the specific plant species under study. In open areas with minimal movement interference, a higher sensitivity can be set, while in more cluttered areas with higher movement levels, a lower sensitivity setting is recommended.

For optimal results, the camera traps should be set at the shoulder height of the target species, as this increases the probability of detection (Palencia et al. 2021). However, in a multi‐species approach, achieving the ideal camera height placement can be challenging. To address this, we suggest using multiple camera deployments to capture a full range of visitors (e.g., ground vs. canopy). In these cases, the field of view of each camera should not overlap to avoid duplication of data. A challenging aspect, almost with any plant species fruiting, is to optimally adjust the field of view while not sacrificing image detail with a deployment at a longer distance. Ideally, individual fruits should be visible in the image of the canopy, under good light conditions, but also with an adequate coverage of the maximum canopy area possible.

We recommend setting the video length consistently to 10–20 s to preserve space in memory cards and batteries and streamline the post‐process workflow. The motion trigger should have no more than 1 s delay to maximize the recording of animal behavior in the scene. The longer length of 20 s, or even longer, might be adequate if obtaining data on fruit foraging and feeding behavior is a relevant objective of the sampling, so that a longer part of the feeding sequence or even the full visit to the plant by the foraging animal can be captured.

Systematically recording a file at the beginning and end of each deployment revision ensures an accurate control of the sampling effort. We offer a code snippet that automates the collection of sampling effort for each camera deployment (see *Code* section below). A summary of the settings recommendations is included in Table S.2.1 in Data S1.

#### 2.2.2 Stage‐2. Processing

CV has demonstrated effectiveness in species recognition and identification, with studies reporting high accuracy rates. For instance, Norouzzadeh et al. (2018) achieved over 99% accuracy and Tabak et al. (2019) reached 97.6% accuracy using recognition algorithms. Yet, automated species identification needs to be further improved and it is essential to be cautious when interpreting model performance, especially when the model training dataset lacks diversity, as this restricts the generalization of the findings to other systems (Greenberg et al. 2019). Attempting to achieve similar accuracies as those provided above may prove unrealistic, particularly when a diverse array of new species need to be identified in an untrained system, as is the case in most plant–animal interaction studies.

##### 2.2.2.1 CV for Removing Empty Videos

Using an object detector model to differentiate between empty and non‐empty videos can save valuable time and is essential for streamlining the analysis process. Most field recordings from plant–animal interaction surveys contain unintended motion triggers caused by the movement of vegetation and thus do not contain relevant information. Removing these blank recordings can considerably reduce the review time. We employ MegaDetector (MD; Beery et al. 2019), an object detection model capable of identifying animals, people, and vehicles in images. Although MD was originally designed for still images, our protocol represents a first attempt to apply object recognition to vast sets of camera trap video recordings.

##### 2.2.2.2 Video Splitting

The implementation of the object detector to video recordings requires splitting the video files into individual frames (Figure 2B). To automate video splitting in large batches, we use an R script (see *Code* section below) that automatically extracts frames from video files and stores them in separate directories maintaining the parent file structure. Although video splitting is time‐consuming, it offers a substantial advantage by significantly reducing the overall file storage size, which is particularly useful when uploading to an external server, a requirement for the use of GPU capabilities.

##### 2.2.2.3 Applying the Object Detection Model

After the video set is split up into individual frames, the model is executed on each individual frame. There are two primary methods to execute the MD object detection model:

1. *Running the model on a local computer*: The model can be freely obtained from the MD GitHub repository (https://github.com/agentmorris/MegaDetector) (Beery et al. 2019). The convenience of running the model locally depends on the number of images to be processed, the computer's hardware specifications, and the user's proficiency in the Python programming language.
2. *Submitting images to MD staff for model execution*: This option is ideal for high‐volume users who require access to high‐performance processors. Whether the model is run locally or by the MD staff, it is advisable to perform a preliminary check by running the model on a few thousand images to ensure the effectiveness on the target dataset.

##### 2.2.2.4 Model Outputs

Once the model is executed a .json file is generated as the main output. This standard data interchange format preserves the inherent structure of the input data, retaining the record of each processed frame file through the frame‐level analysis. This frame‐level output includes the probability, or confidence score, at which the model detects the presence of an animal (also person and vehicle, which are discarded for our specific purposes) in each frame. To obtain the video‐level output, we aggregate the frame‐level results for each video file by selecting the highest confidence value from the set of frames (Figure 2B). This highest confidence value corresponds to the model's confidence in detecting the presence of an animal in the video.

##### 2.2.2.5 Visualization and Database Creation

Time‐lapse software (Greenberg 2020), an open‐source tool designed for reviewing camera trap images including videos facilitating the creation of ecological‐interaction datasets. It supports video analysis with tools that enhance visual encoding and data entry efficiency (Figure 2C). The software automatically extracts video metadata such as footage time and RelativePath and it offers a customizable interface for data input, supporting visual searches. Time‐lapse integrates seamlessly with image recognition pipelines (e.g., MegaDetector output), allowing users to filter data by object detection confidence levels, excluding empty videos and optimizing workflows. Customizable templates aligned with the “Observations.csv” data structure (see Supporting Information S1 in Data S1 for detailed information) simplify tagging information such as species, counts, and behaviors.

For species identification, an expert field ecologist visually reviewed the videos, using anatomical characteristics and taking advantage of the video format, which provided movement cues that are especially helpful for distinguishing between similar species. If the expert was unable to confidently identify the species, the video was revised by four to six additional experts, each providing an independent assessment. When at least three of them agreed on a species identification, that consensus was accepted. If no consensus was reached, the observation was classified as unidentified and kept out of further analysis. Animal behavior was also annotated, including activities such as foraging, perching, walking, or simply passing through. Behaviors related to foraging were particularly relevant for documenting frugivory interactions. To reflect this, we defined a hierarchy of feeding behaviors: “feeding” *sensu stricto* was used when the animal was clearly observed ingesting fruit; “probably feeding” was applied when the fruit was being handled but ingestion was not visible in the frame; and “searching for food” referred to cases where the animal appeared to be looking for fruit, even if the fruit itself or the act of feeding was not captured in the frame. These last cases included: (1) foraging actively for fruits, with head movements directed to fruit seeking; (2) body orientation or postures indicative of fruit foraging; (3) perching positions close to infructescences and movement/positions indicative of active foraging. Thus, we discarded as negative records those with animals passing by the fruiting branches, or stationary perching positions in the case of birds, chasing movements, or any other foraging maneuvers not indicative of fruit‐feeding. While direct observation of fruit handling/ingestion is ideal for a positive record of an interaction, it is rarely visible in video footage, especially in field conditions and when using camera traps. The limited field of view provided by camera traps captures only a fraction of the interaction space, often missing critical behaviors occurring just outside the frame. In this context, inferring feeding behavior based on foraging, fruit picking, handling, or searching patterns is both necessary and consistent with established ecological methodologies (e.g., Moermond and Denslow 1985; Levey 1987; see also Da Silva and Dos Reis 2019). Otherwise, a bias underestimating actual PIEs would have been introduced if these records with active fruit foraging are discarded. The final output is a plain text (.csv) file listing videos with identified animals, their behaviors, and metadata (Figure 2D). This functionality makes Time‐lapse an efficient solution for ecological research requiring detailed behavioral and species observations.

#### 2.2.3 Stage‐3. Post‐Processing

##### 2.2.3.1 Database Handling

The dataset comprises 10 s video recordings capturing one or more animals exhibiting various behaviors, with a focus on fruit consumption in the context of frugivory (Figure 2D). A conservative approach would involve selecting only videos showing animals directly handling or ingesting fruit. However, this method risks underestimating fruit‐feeding and interaction frequencies by discarding instances where feeding behavior is implied but not explicitly visible, as discussed above. Such cases may occur when the animal's actions are obscured or fall outside the camera's view during the short recording duration. To address this limitation, it is recommended to include videos where animals are likely engaged in feeding bouts or actively searching for food, based on expert assessment. This broader inclusion ensures a more accurate representation of interaction frequencies and feeding behaviors within the dataset, avoiding underestimations of *PIEs*.

##### 2.2.3.2 Video Collapsing

To mitigate bias caused by temporal autocorrelation in consecutive 10 s video recordings, it is essential to define unique frugivory interaction events using an objective criterion. This protocol establishes a 5 min duration threshold to create independent events (Figure 2E). A frugivore visit begins when the animal arrives at a plant and ends upon departure, with visits often exceeding 10 s. During these visits, fruit handling or ingestion may be recorded, though complete visit durations are rarely captured. Pooling successive videos within 5 min intervals effectively represents a single interaction, decreasing the likelihood that distinct visits by different individuals are confounded (aggregated) into one event. This method balances the need for sufficient differentiation between visits while accommodating the typically short duration of interactions. By adopting this approach, the analysis avoids introducing bias and ensures a more accurate representation of frugivory behavior.

##### 2.2.3.3 Sampling Effort

Sampling effort recording is crucial for controlling the level of effort for the different deployments, plant individuals and plant species, allowing an analysis and comparison of the amount of time spent on each of them, in order to evaluate the data collection process. Sampling effort information is automatically extracted from the parent directories at the video level by an ad hoc code. The code relies on the metadata from the video files from a camera recorded during each revision, which are stored with this structure. As previously mentioned, triggering a first and last recording as explained in the Pre‐Processing section is mandatory to ensure accurate sampling effort information. The code calculates the duration of each deployment based on the date and time of the first and last video, which represents the sampling effort for a specific deployment (Figure 2E). The duration, expressed in decimal numbers (days), can be aggregated for individual and species levels and used to estimate accumulation curves for interaction records (see e.g., Villalva et al. 2024; Quintero et al. 2022).

#### 2.2.4 Code

The code functions provided in our streamline protocol (https://github.com/PJordano‐Lab/Ecological‐interactions‐camtrap‐protocol/releases/tag/v1.0.0) include five main tasks.

- *Video splitting*: Automates the process of recursive extraction of frames from video files. It organizes the frames into new directories following the same structure as the original ones, and is used for running the image detection model at frame level.
- *Working with json*: Processes JSON data, filters and selects specific video files based on their confidence levels, moves the selected video files to new directories with modified paths, and combines data from multiple JSON files into a single data frame as input for the visualization software.
- *Video collapsing*: Collapses video events in fixed intervals from the ‘DateTime’ values, and performs various aggregations and calculations for each group.
- *Obtaining sampling effort*: Processes video files from parent directories, calculates the duration and number of files for each subdirectory, organizes the results into a data frame, manipulates the data frame to extract relevant information, and writes the processed data to a .csv file for further merging with the video‐level dataset.
- *Obtaining file metadata*: Reads and processes video files' metadata (e.g., video duration and creation time) from the parent directory, to merge the resulting metadata to the video‐level dataset (e.g., EXIF tools).

### 2.3 Validation Method

To assess the effectiveness of CV and evaluate data loss in its application, we used the recently compiled Frugivory‐camtrap database, which offers a comprehensive overview of frugivory interactions within Doñana National Park (Villalva et al. 2024). The database was constructed using the workflow outlined in this paper. For a comprehensive understanding of the dataset's structure, scope, and quality, we refer readers to the accompanying metadata provided with the database (Villalva et al. 2024). Due to the clear logistic and time limitations for visualizing the entire video set (including FN) in order to evaluate the performance of CV, we randomly selected 10% (*n* = 14,123 videos) out of the raw files for the full 2021–2022 season (total sample size, *n*_*t*_ = 138,351 videos; sampling effort = 7184 camera trap‐days). This subset was stratified to maintain the number of videos proportional to the total number of videos per species, allowing us to assess binary classifications and build confusion matrices based on an established confidence threshold of 0.8 (a conservative value for MDv4). We computed fundamental performance metrics from the confusion matrices for each species (Table 2). Additionally, we incorporated performance indicators such as the F Score, widely used in statistical ecology. The F Score is the harmonic mean of precision (the proportion of positive identifications that were actually correct) and recall (the proportion of actual positives that were correctly identified), and it is particularly useful when evaluating the trade‐off between false positives and false negatives (Chinchor 1992). The MCC, on the other hand, takes into account all four elements of the confusion matrix (true positives, true negatives, false positives, and false negatives), providing a balanced evaluation even when the classes are of very different sizes. It is especially recommended for use with imbalanced datasets (Chicco and Jurman 2020), where one class (e.g., videos with animal interactions) is much rarer than another (e.g., empty videos). Confusion matrices were generated for each focal plant species and individual plant, allowing us to examine the information loss by analyzing FN and identifying potential reasons for detection failures, combining success, TP and FN estimates.

**TABLE 2:** Common statistical rates and performance metrics to evaluate binary classifications through their confusion matrices.

Acronym	Description	Equation
TPR	True positive rate or Sensitivity (Recall)	TP/(TP + FN)
TNR	True negative rate or Specificity	TN/(FP + TN)
FPR	False positive rate or Fall out	FP/(FP + TN)
FNR	False negative rate or Miss rate	FN/(FN + TP)
PPV	Positive predictive value or Precision	TP/(TP + FP)
NPV	Negative predictive value	TN/(TN + FN)
FDR	False discovery rate	FP/(FP + TP)
FOR	False omission rate	FN/(FN + TN)
ACC	Accuracy	(TP + TN)/(TP + TN + FP + FN)
ERR	Error rate	(FP + FN)/(TP + TN + FP + FN)
F1 Score	Harmonic mean between TPR and PPV	(2*TP)/((2*TP) + FP + FN)
MCC	Mathews correlation coefficient	((TP*TN)‐(FP*FN))/ sqrt((TP + FP)*(TP + FN)*(TN + FP)*(TN + FN))

Abbreviations: FN, false negative; FP, false positive; TN, true negative; TP, true positive.

To evaluate the potential impact of FN on the inferred network structure, we conducted a direct comparison between the two bipartite interaction networks derived from the video data with and without including FN. The first network included confirmed records of interaction events (*n* = 10,659 videos with fruit ingestion and/or fruit picking and handling visualization), while the second incorporated all available data by imputing FN based on the human revision of the 10% stratified random sample (*n* = 11,574 videos). Both networks were represented as adjacency matrices (Mt and Mfn, respectively), and the corresponding graph objects (*gt* and *gfn*) were compared using Quadratic Assignment Procedure (*QAP*) tests implemented in the *qaptest* function from the R package *sna* (Butts 2024). We assessed the similarity between graphs by randomly permuting node labels across a large number of iterations and evaluated the proportion of permuted graphs with test parameters as extreme as the observed. We used Hamming distance (*Hd*) and graph correlation (*gcor*) as structural comparison metrics (Butts and Carley 2005).

## 3 Results

The primary output resulting from our data analysis is the streamline protocol itself, which establishes a validated workflow facilitating the integration of CV to video recordings, addressing several crucial aspects such as data structuring and the incorporation of sampling effort, which are elaborated upon in the methods section (Figure 2), as well as the accessory code useful for implementing the workflow that can be used as a tool.

Upon visualizing and analyzing the randomly stratified sample subset, the overall confusion matrix was obtained (see Table 2 and Supporting Information S3 in Data S1 for a conceptual summary of the confusion metrics). A confidence threshold of 0.80 resulted in the loss of 767 video clips containing animals. Out of this total, 81 clips were indeterminable due to various factors rendering them invisible in the footage (e.g., unknown species), not directly affecting our objective of gathering data for ecological interactions. Notably, 48% of the missing clips (*n* = 367) depicted various foraging behaviors (such as feeding, probable feeding, and searching for food), therefore representing legitimate missing data that directly influence data gathering for our purpose. Specifically, 83 clips depicted animals clearly engaged in fruit handling and feeding behaviors, while half of these animals were in the process of searching for fruit. The overall confusion matrix for the model (confidence level > 0.8) showed an accuracy of 0.92. However, there was significant variability in accuracy across different plant species ranging from 0.85 to 0.97 (Table 3, Figure S.3 in Data S1).

**TABLE 3:** Confusion matrices, species‐specific and combined, displayed in tabular format for a designated confidence threshold (*c* = 0.80) to depict how the CV model's performance varies across various species (figures refer to the proportion of cases—no. of videos—falling in each category indicated as column names).

Plant species	True positive	True negative	False positive	False negative	No. videos
*Arbutus unedo* (10)	0.09	0.80	0.00	0.10	1431
*Asparagus aphyllus* (8)	0.14	0.73	0.06	0.07	525
*Corema album* (24)	0.05	0.92	0.01	0.02	2087
Juniperus macrocarpa* (13)	0.08	0.87	0.01	0.04	391
*Myrtus communis* (9)	0.01	0.96	0.00	0.03	936
*Olea europaea* (5)	0.31	0.57	0.01	0.12	729
*Pinus pinea* (5)	0.75	0.10	0.02	0.13	534
*Pyrus bourgaeana* (12)	0.17	0.75	0.03	0.05	2670
*Rubus ulmifolius* (27)	0.03	0.89	0.02	0.07	3357
*Smilax aspera* (6)	0.01	0.95	0.02	0.02	1463
All species (119)	0.11	0.8	0.02	0.06	14,123

*Note:* No. of videos refers to the size of a stratified random sample of the dataset that were visually revised (*n =* 14,123), without CV assistance; such a sample was extracted from the total of *n* = 138,351 videos obtained. In brackets, number of sampled individuals. * *J. macrocarpa var. oxycedrus*.

**FIGURE 3:**
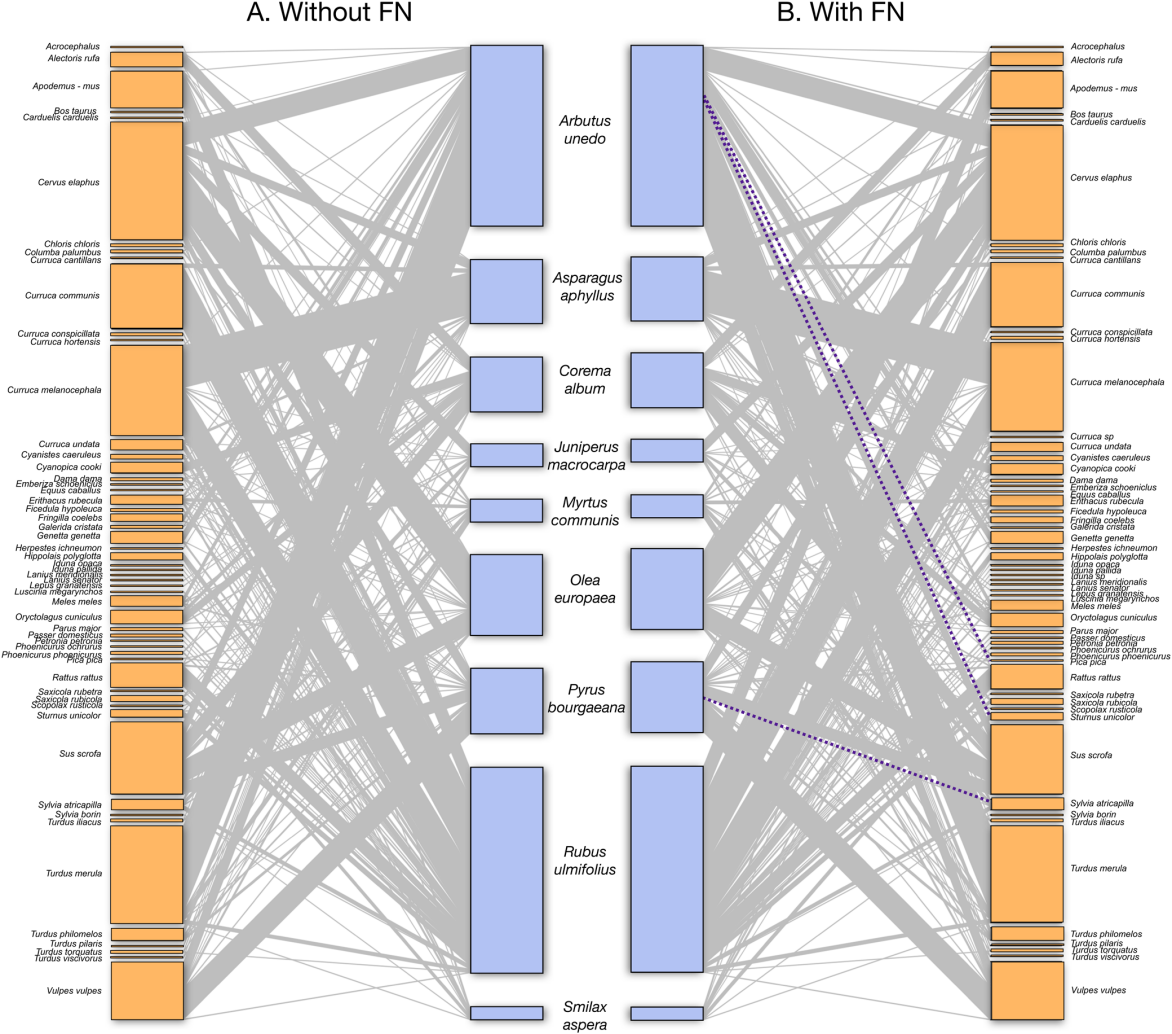
Community‐level network built using CV (left) and with the inclusion of false negatives (right). Unique pairwise interactions missed by applying CV are highlighted in purple dotted lines. The size of the rectangles representing plants (blue) and animals (orange) nodes reflects the frequency of events involving each individual or species respectively. The thickness of connections indicates the relative frequency of each pairwise interaction.

The degree of missing information varied among species, with pairwise interactions declining by 1 to 10%. For example, in two distinct plant species‐ *Arbutus unedo* and *Pyrus bourgaeana‐* the model's failure also resulted in the loss of three unique interactions (Table 4, Figure 3). Correspondingly, performance metrics exhibited variability; certain species (e.g., *Olea europaea* and *Pyrus bourgaeana*) demonstrated high performance and consistent CV behavior, whereas others (e.g., *Smilax aspera* ) performed poorly.

**TABLE 4:** Outcomes derived from video recordings capturing ecological interactions assisted by CV (RAW data) and the subsequent validation of a stratified random 10% of the dataset (information lost).

Plant species	Raw data (network)		Lost information (Pairwise Interactions)		Lost information (Unique interactions)	
	No. interactions	No. species	Interactions lost	Species lost	Interactions lost	Species lost
*Arbutus unedo*	2229	24	64 (2.9%)	12	2	*P. pica* (1) *S. unicolor* (1)
*Asparagus aphyllus*	777	13	16 (2.1%)	4	0	—
*Corema album*	656	18	13 (2.0%)	7	2	Curruca sp* (1) *C. undata* (1)
*Juniperus macrocarpa*	270	9	3 (1.1%)	2	0	—
*Myrtus communis*	257	12	12 (4.7%)	6	0	—
*Olea europaea*	973	20	30 (3.1%)	7	0	—
*Pyrus bourgaeana*	806	16	75 (9.3%)	11	2	*S. atricapilla* (1) Curruca sp* (1)
*Rubus ulmifolius*	2553	37	116 (4.5%)	14	0	—
*Smilax aspera*	150	11	15 (10.0%)	4	0	—

*Note:* Results are grouped by focal plant species (rows) in three sets of columns detailing the (i) total number of interaction events recorded, as supported by CV (excluding absent interactions), (ii) loss of information concerning pairwise interactions (number of interactions and species lost) and (iii) the loss of unique interactions (number of unique interactions and abbreviated name of species lost). Unique species lost: *Pica pica; Sturnus unicolor, Curruca undata; Sylvia atricapilla*. *Curruca sp. could belong to any species within the *Curruca* genus, yet it was not possible to discern the specific species from the video clip. The RAW dataset was constructed specifically for fleshy‐fruited species, which is why *Pinus pinea* was excluded from this table.

We were unable to discern the cause of failure in 29% of cases. However, we were able to determine that the majority (38%) of undetected species were obscured by vegetation in the background of the scene, likely eluding the CV's detection. Interestingly, approximately 12% of species were overlooked by the CV probably due to their mimetic characteristics, such as the case of *Carduelis carduelis* in *Arbutus unedo* , where a substantial loss of interactions (90% of this particular pairwise interaction) occurred likely because its colors resembled the prevalent yellow‐red tones in *Arbutus unedo* . A total of three unique pairwise interactions were lost when using this protocol, namely *Arbutus unedo–Pica pica*, *Arbutus unedo–Sturnus unicolor* and *Pyrus bourgaeana–Sylvia atricapilla* (Table 4, Figure 3). All three interactions involving animals in search for food were missed because the animal was located in the background of the scene.

We carried out a direct comparison of the structure of the two networks resulting from the visualization protocol: one including the data with direct records of fruit handling/feeding and active fruit foraging (*n*_*t*_ = 10,659 videos), and another including all the information, by imputing the potential FN (*n*_*fn*_ = 11,574 videos). For both statistics, *H*_*d*_ and graph correlation, we obtained no evidence that the subsampled networks (without *FN* imputation) would result in structurally different network topologies than the networks including imputed *FN*s: *H*_*d*_ = 831, *P < <* 0.0001, significantly lower than expected for different networks; and *gcor* = 0.9817, *P < <* 0.0001, suggesting a marked graph correlation between the two networks. Therefore, even ignoring *FN*s we could still accurately represent the structural properties of these complex networks; in other words, imputing *FN*s does not appear to overestimate or otherwise alter the link distribution and network structure of actual fruit‐handling records in the overall community network.

## 4 Discussion

This protocol streamlines the creation of datasets for analyzing plant–animal interactions and other animal behavior studies requiring field‐based video recordings, as we exemplified using a study case with plant‐frugivore interactions for seed dispersal. By applying computer vision to classify videos with or without animals, it significantly reduces the time and effort needed to process large volumes of data. In a pilot evaluation, the workflow reduced video annotation time from three weeks to three days, cutting effort by 1/7 while producing robust interaction data with minimal variation. While we did not evaluate the economic cost of applying this method directly, previous studies have shown that developing automated workflows for wildlife camera trapping can be cost‐efficient (Kissling et al. 2024). However, the use of CV introduces some information loss, particularly when animal species or behaviors (e.g., foraging) are undetected by the CV model. While much of the lost data was deemed irrelevant to building ecological network databases, some relevant interactions were missed, potentially biasing results. This loss was uneven across species and plant individuals, influenced by factors like vegetation cover, lighting conditions, and fruit visibility. Missing interactions could influence key ecological metrics such as interaction strength, network stability, resilience, and connectivity. For functionally unique species, those serving as hubs, keystone resources, or specialists, false negatives may lead to misclassification of their ecological roles and affect our interpretation of network structure and stability. If interactions involving unique species are systematically underdetected, this could lead to their undervaluation in conservation planning or ecosystem restoration strategies. Moreover, even a small proportion of missed interactions (e.g., < 10%) could represent rare events with high ecological significance, such as dispersal by large‐bodied frugivores with long‐distance foraging ranges potentially contributing to long‐distance dispersal events. These limitations highlight the need for caution when inferring network completeness solely from automated detection. To mitigate these potential biases, replication of camera trap locations is recommended to capture diverse environmental conditions. Adequate sampling effort across the fruiting phenophase and careful camera placement (avoiding obstructions like branches or excessive shade) are also critical. While these findings were based on the MD model, similar issues may arise with other object detection models, underscoring the need to validate CV tools within specific ecological contexts to ensure accuracy.

**FIGURE 4:**
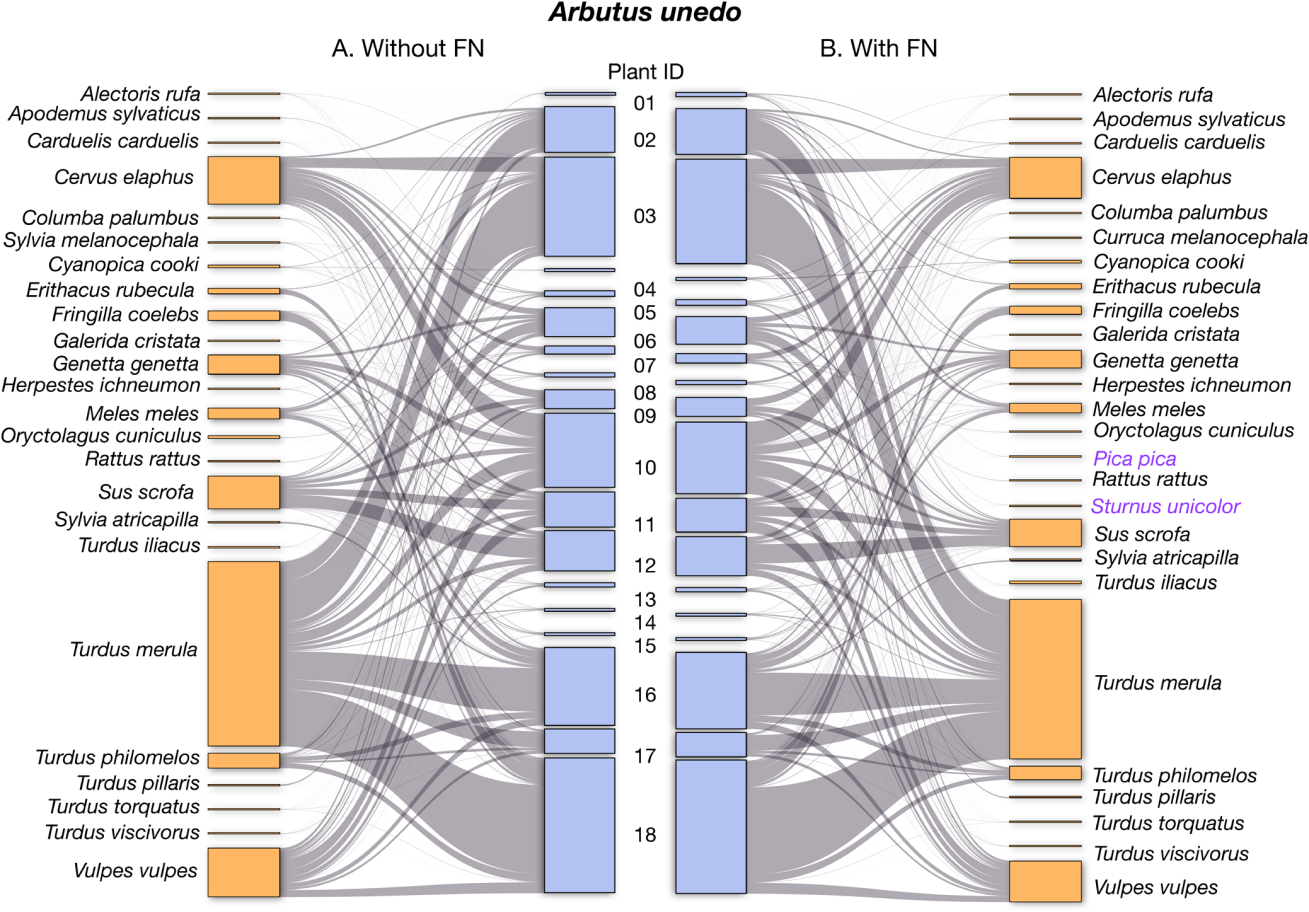
Individual‐based network of plant–animal interactions including false negatives (right) contrasting with one constructed without false negatives (left). Individual plants of *Arbutus unedo* are represented as blue rectangles positioned in the center (numbers identify individual plants sampled). Animal species nodes are aligned at the margins, depicted as orange rectangles. The size of the rectangles reflects the frequency of events involving each plant individual or animal species. The thickness of connections (gray) indicates the relative frequency of each pairwise interaction. Animal species common to both networks are depicted in black, while those present in one network but not the other are highlighted in purple.

The loss of information caused by CV is not easy to eradicate. In many cases, animal species were present in the scene, but located in the background and only recognizable by movement patterns, size, sound, or by context‐dependent and species‐specific characteristics that can be only appreciated by human‐expert curation at this point of the state of the art in CV. Currently, these contingencies are difficult to emulate through CV models and it would be unrealistic trying to avoid them regardless of how detailed the training dataset is. In some cases, the loss of data was attributed to the mimicry of certain animal species on specific plant species. For example, in the interaction of *Arbutus unedo* and *Carduelis carduelis* , facial and wing colors (red, cream, black, and yellow) closely resemble the reddish‐orange tones of the ripe fruits, resulting in a 90% loss of information for this particular interaction. This phenomenon, clearly evident in this species, may be happening among species with more cryptic plumage such as certain thrush species (e.g., *Turdus philomelos* ), which may go unnoticed especially when standing on open ground compared to *Turdus merula* , generally much more visible, potentially biasing the database. In any case, in our experience, those instances occurred very infrequently and, again, replication and sufficient sampling effort may counteract potential biases. Although the interactions between *Carduelis carduelis* and *Arbutus unedo* were largely missed, mainly affecting a single individual (see Figure 4, plant individual 04), the interaction was still captured in other instances and thus recorded overall in the community network (Figure 3). More concerning, however, is the potential loss of rare interactions, which are characterized by their low frequency of occurrence. In our validation analysis, we identified only three such lost unique interactions (Figure 3), involving *Sylvia atricapilla* , *Sturnus unicolor* , and *Pica pica* , all of which are common species in the area that likely interact only occasionally with *Pyrus bourgaeana* or *Arbutus unedo* . This suggests that while some rare events may go undetected, the overall impact of information loss on community‐level assessments is limited, as further supported by our network structure analysis.

Our results demonstrate the effectiveness of using CV‐based methods to construct community‐level species interaction networks, with minimal information loss relative to the extensive data gained. Previous studies (Villalva et al. 2024; Quintero et al. 2024; Isla et al. 2024) confirmed sufficient sampling coverage and accurate interaction frequency estimation using camera‐trap methods. The automated CV‐validated network structure closely mirrors non‐automated results, ensuring reliability (Figure 3). However, some unique interactions, particularly those involving less common frugivores, may be overlooked, potentially impacting zoocentric analyses of animal frugivory and fruit resource use. From a phytocentric perspective, however, these losses are less critical since dominant seed dispersers play key roles in maintaining fleshy‐fruited tree species' persistence within the broader frugivore community. When constructing networks at the individual level (i.e., documenting frugivore species interacting with different plant individuals in a local population, e.g., Quintero et al. 2024), information loss was not evenly distributed, being more pronounced in specific individuals which may systematically offer biased data. The impact of interaction loss on certain individuals can substantially influence analysis outcomes, especially if the data are intended to be used for individual‐based approaches (based on the variability among individuals). To mitigate this potential bias in individual‐based approaches, employing additional complementary methods alongside camera traps, such as direct observations, mist netting, or scat barcoding, can provide a more comprehensive inventory of individual assemblages (Quintero et al. 2022), together with increasing sampling effort for particular plant individuals.

**FIGURE 5:**
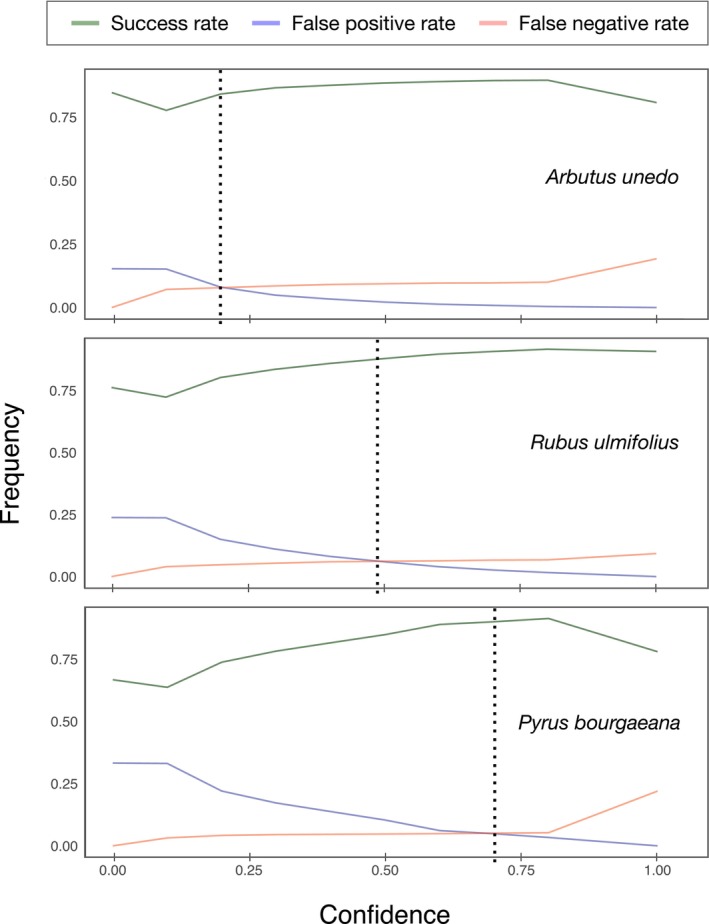
Cumulative curves depicting success (green line), false positive (red) and false negative (blue) rates relative to confidence level for three plant species, highlighting potential confidence thresholds. A dotted vertical line indicates the species‐specific confidence threshold, where false positives and false negatives are minimized, while ensuring a high success ratio.

Our study highlights the complexity of selecting an appropriate confidence threshold for reviewing video data, particularly when large unbalanced datasets make lowering confidence levels impractical. Ensuring data robustness requires minimizing the effort in viewing and tagging videos while maintaining data integrity. Our results indicate that there is no universal confidence threshold applicable to all scenarios. Instead, threshold selection must be tailored to specific contexts, taking into account factors such as the characteristics of the focal plant, background complexity, and habitat conditions. Effective threshold selection involves carefully balancing the false negative rate (missed sequences containing relevant interactions) with the false positive rate (empty videos that require unnecessary manual review), in order to optimize both efficiency and data accuracy. As model confidence increases, the false negative rate decreases while the false positive rate rises, making it necessary to identify an optimal threshold that balances both (Figure 5). To address this, we encourage users to perform their own calibrations by visualizing a random subsample of videos to construct a confusion matrix and plot cumulative curves of FP and FN rates across confidence levels. This approach identifies the optimal threshold that minimizes both rates. Crucially, the results demonstrate that optimal confidence levels vary by plant species, ranging from approximately 0.25 to 0.75. This is not at all unexpected, considering the ample diversity of growing form, habit, infructescence structure, background vegetation cover, physiognomy of the growing site, etc. specific to each plant species. Applying a uniform confidence threshold across species—a common but flawed practice—can introduce significant biases, leading to uneven information loss among species. The study underscores the importance of obtaining species‐specific confidence thresholds, determined through preliminary assessments using random subsamples of video data (Figure 2F). This variation likely stems from differences in plant structure and background, as noted by Palencia et al. (2021), and may also occur in other contexts, such as animal detection studies (e.g., Vélez et al. 2023). The findings emphasize the need for tailored approaches to optimize CV‐based video analysis in ecological research.

**FIGURE 6:**
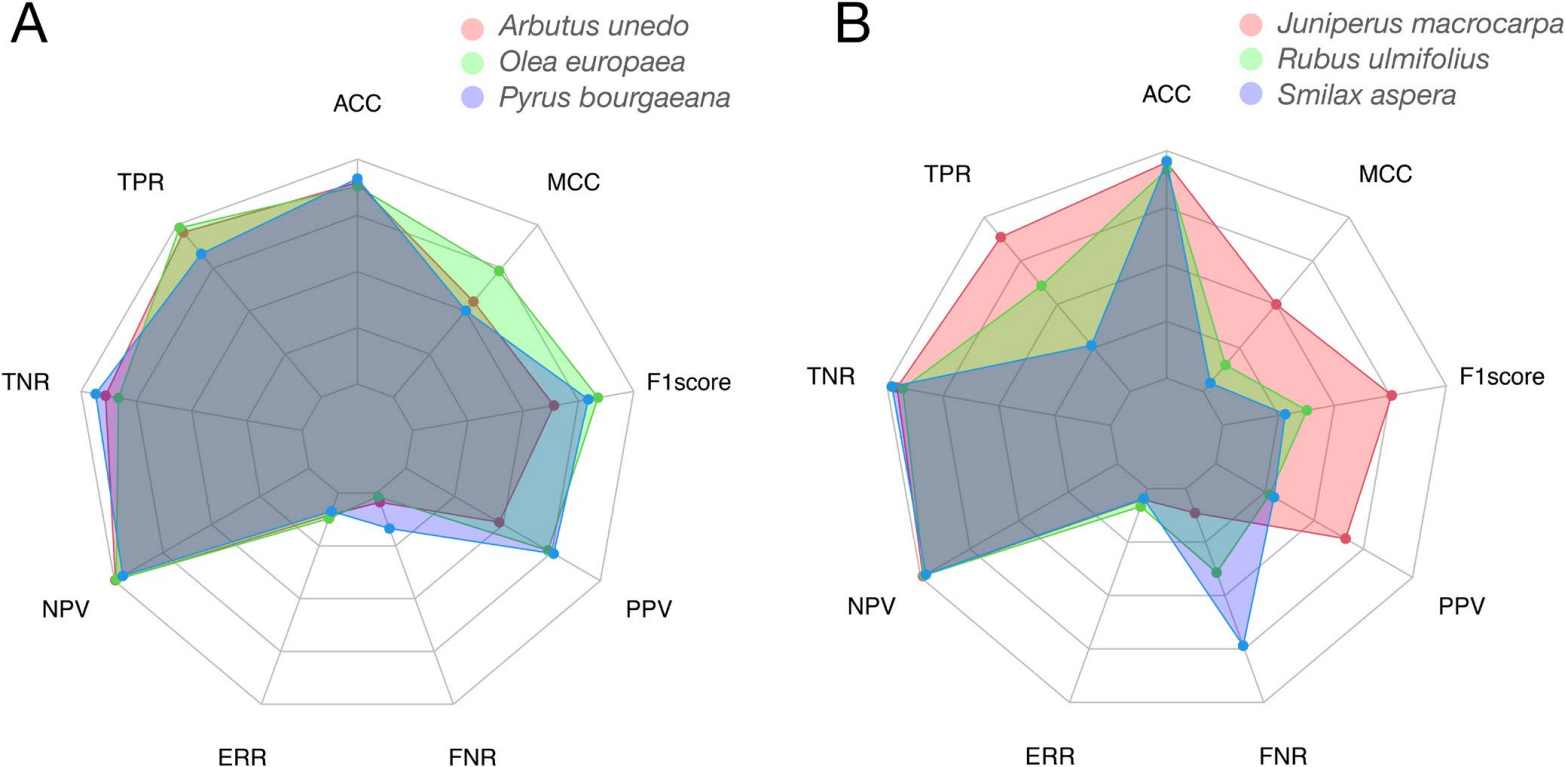
Performance metrics for six distinct plant species, represented by different colors in spider web plots, evaluated for nine different metrics (radii in the plots). On the left panel, three species exhibit consistent and satisfactory performance metrics, whereas the right panel illustrates three species with varying performance, including two with poor results. Performance metrics used: [TPR] True Positive Rate (Sensitivity, Recall), [TNR] True Negative Rate (Specificity), [FPR] False Positive Rate (Fall Out), [FNR] False Negative Rate (Miss Rate), [PPV] Positive Predictive Value (Precision), [NPV] Negative Predictive Value, [FDR] False Discovery Rate, [FOR] False Omission Rate, [ACC] Accuracy, [ERR] Error Rate, [F1score] F1 Score (Harmonic mean between TPR and PPV), [MCC] Matthews Correlation Coefficient.

The application of image recognition to camera trap data has predominantly focused on accuracy as the primary predictive metric (e.g., Norouzzadeh et al. 2018; Tabak et al. 2019). However, this approach is insufficient for assessing data quality in certain contexts. Accuracy can be misleading (Greenberg 2020), particularly with unbalanced datasets where one class (often negatives) dominates, resulting in an overly optimistic evaluation of a model's performance. This issue also extends to the F1 score, which is similarly inadequate for unbalanced data. Instead, the Matthews Correlation Coefficient (MCC) is proposed as a more suitable metric for such scenarios (Chicco and Jurman 2020). Unlike accuracy and F1, MCC accounts for class imbalance and evaluates both positive and negative classifications comprehensively. It offers a more reliable and informative measure of model performance, particularly in binary classification tasks (Figure 6). MCC has already been successfully applied in genomic studies, demonstrating its utility in contexts where balanced evaluation across classes is critical. Thus, MCC emerges as a robust alternative for evaluating CV models on unbalanced datasets.

Image recognition models continue to be enhanced with new data, leading to better performance over time. For instance, the newer version of MegaDetector (MDv5) increased processing speed and incorporated additional training data to improve the detection of particular taxa (particularly rodents, reptiles, and small birds). These updates, along with advancements in CV platforms for data management and integration, will further enhance the efficiency of ecological studies in the near future.

Amid escalating biodiversity loss, efforts to mitigate human impacts are intensifying, exemplified by the 23 targets of the 2022 Kunming–Montreal Global Biodiversity Framework. While numerous Essential Biodiversity Variables (EBVs) exist, many oversimplify biodiversity by focusing on species or habitat trends, neglecting ecosystem complexity and ecological functions critical for conservation. Standardized protocols integrating advanced technologies offer significant potential for large‐scale applications, such as national assessments of community structure. These methods provide valuable insights into biodiversity changes, ecological interactions, and functions, aiding in quantifying biodiversity loss and restoring ecological processes. Broad‐scale implementation could be enhanced by combining these approaches with remote sensing to analyze vegetation colonization fronts and their links to biodiversity complexity. Such advancements are pivotal for comprehensive biodiversity monitoring and the development of effective conservation strategies.

Our protocol optimizes the collection of robust field data for analyzing plant–animal ecological interaction networks through a standardized workflow. Building on camera trap standards and CV‐powered platforms, it provides detailed guidelines for camera setup, data organization to automate sampling effort, and computer vision techniques for classifying videos with or without animals. The approach streamlines data processing, producing datasets ready for network analysis or behavioral studies while reducing time and effort. It also includes methods to determine confidence thresholds for focal species, balancing FN and FP, and addresses limitations of CV in individual‐based analyses, emphasizing its reliability for community‐level studies. Key challenges and potential caveats are critically discussed.

## Author Contributions

**Pablo Villalva:** conceptualization (equal), data curation (lead), formal analysis (equal), funding acquisition (supporting), investigation (equal), methodology (equal), project administration (supporting), resources (lead), software (lead), supervision (equal), validation (equal), visualization (equal), writing – original draft (equal), writing – review and editing (equal). **Pedro Jordano:** conceptualization (equal), data curation (supporting), formal analysis (equal), funding acquisition (lead), investigation (equal), methodology (equal), project administration (lead), resources (supporting), software (supporting), supervision (lead), validation (equal), visualization (equal), writing – original draft (equal), writing – review and editing (equal).

## Funding

This work was supported by Ministerio de Ciencia, Innovación y Universidades‐MICIU/AEI/10.13039/501100011033/, PID2022‐136812NB‐I00. LifeWatch ERIC–SUMHAL–FEDER–EU530, LIFEWATCH–2019–09–CSIC–13. Center for Sustainable Landscapes under Global Change, Naturmål and SustainScapes–NNF20OC0059595. Universidad de Sevilla, Plan Propio de Investigación y Transferencia, US.

## Conflicts of Interest

The authors declare no conflicts of interest.

## Supporting information


**Data S1:** ece372584‐sup‐0001‐supinfo.pdf.

## Data Availability

Data and code are available at: https://github.com/PJordano‐Lab/Ecological‐interactions‐camtrap‐protocol/releases/tag/v1.0.0 as release v.1.0.0 and https://zenodo.org/records/17353294. Data for analysis comes from: Data available from the CSIC Open Access repository: https://doi.org/10.20350/digitalCSIC/15623 (Villalva et al. 2024 [https://doi.org/10.1002/ecy.4424]).
