# Supplementary material for: A Machine Learning Application to Camera‐Traps: Robust Species Interactions Datasets for Analysis of Mutualistic Networks
Source: Ecol Evol. 2026 Jan 21;16(1):e72584. doi: 10.1002/ece3.72584 (PMC12823160; doi:10.1002/ece3.72584)
Supplement: Supplementary file 1 — Data S1: ece372584‐sup‐0001‐supinfo.pdf. [file ECE3-16-e72584-s001.pdf]

Supplementary Materials

A Machine Learning Application to Camera-Traps: Robust Species Interactions Datasets for Analysis of Mutualistic Networks

Pablo Villalva[1,2], Pedro Jordano[2,3]

[1], Center for Sustainable Landscapes under Global Change. Dept. of Biology, Aarhus University, Aarhus, Denmark.
[2], Integrative Ecology Group. Estación Biológica de Doñana, CSIC. Sevilla, Spain.
[3], Dept. Biología Vegetal y Ecología, Universidad de Sevilla, Sevilla, Spain.

S.1. *Database structure*

A field sampling using camera traps for recording ecological interactions most likely involves the simultaneous deployment of multiple cameras, in replicated positions to target different individual plants, throughout the fruiting season of different focal plant species. The cameras are checked at regular intervals, typically weekly, biweekly, or monthly, which can result in a large number of videos with the same name and date (see e.g., Villalva et al. 2024).

Effective management of such a large and complex data set is crucial for a successful database creation. To achieve this, a structured field database is required to keep track of the data at every stage of the process. For this purpose, we recommend the use of the Camera Trap Data Package (Camtrap DP) (Desmet et al 2021, Bubknicki et al. 2023), a community-developed data exchange format that is under development as a Biodiversity Information Standard (TDWG) (https://camtrap-dp.tdwg.org). This package provides a useful structure for controlling camera-trap data at three levels, from which we will adopt the structure: *deployments*, *revisions*, and *observations*.

Camtrap DP offers a standardized format for organizing camera-trap data, ensuring consistency and reducing the risk of errors or inconsistencies. This structure includes all necessary information, such as camera settings, deployment locations, and video file names, allowing for easy management and analysis of the data. A template for the Camtrap DP is available for use, and you can find a template for our ad-hoc structure in the GitHub repository (https://github.com/PJordano-Lab/Ecological-interactions-camtrap-protocol/tree/main/Preprocess) or see the following descriptions.

The main data is structured in three related plain text files (.csv) as follows:

File	Description
deployments.csv	Table with camera trap deployments.
video.csv	Table with media files captured by the camera traps.
observations.csv	Table with observation records based on media files (after viewing in Timelapse).

Table S.1.1 ***Deployments***

Table with camera trap deployments. Includes deploymentID (focal species acronym + cameraID), Location, and camera Setup information for each camera.

Name	Definition	Type
Deployment_ID	Unique identifier of the deployment. Name of the focal species followed by the individual number low dash camera number.<br><br>Example: Aune003_58	string
Location	Name given to the deployment location. Survey area.	string
Longitude	Longitude of the deployment location in decimal degrees, using the WGS84 datum.	number
Latitude	Latitude of the deployment location in decimal degrees, using the WGS84 datum.	number
Start	Date and time at which the deployment was started. Formatted as an ISO 8601 string with timezone designator.<br><br>(YYYY-MM-DDThh:mm:ss±hh:mm).	datetime
End	Date and time at which the deployment was ended. Formatted as an ISO 8601 string with timezone designator. (YYYY-MM-DDThh:mm:ss±hh:mm)	datetime
Days	Number of days the deployment was set in the field. End_date - Start_date	number
Setup_by	Name(s) or unique identifier of the person that deployed the camera.	string
Camera_ID	Unique identifier of the camera used for the deployment (could be the serial number but also a simple number).	string

Camera_model	Manufacturer and model of the camera.	string
Comments	Comments or notes about the deployment.	string

Table S.1.2 *Videos*
Table with video files captured by camera traps. Associated with deployments (by deploymentID) and organised in revisions (revision_ID). Includes Timestamp_Issues and File_path.

Name	Definition	Type
Deployment_ID	Unique identifier of the deployment the media file belongs to. Foreign key to Deployments.Deployment_ID	string
Revision_ID	Unique identifier of the revision the media file belongs to. Revisions contain one or more media files (e.g. a single image or video or a sequence of successive images or videos). Example: Rev_01	string
Videos	Number of media files.	number
First_video	Datetime for the first video in the revision sequence.	daytime
Last_video	Datetime for the last video in the revision sequence.	daytime
Days	Number of days that the deployment was set in the field during the current revision.	number
Setup_date	Date at which the camera was set on in the current revision. Date at which the camera was set off in the current revision.	daytime
Revision_date	Date at which the camera was set off in the current revision. Date at which the camera was set off in the current revision.	daytime
Functioning_days	Number of days where the camera was functioning. Revision_date - Setup_date	number
Battery	Percentage of battery in the revision datetime,	string

Timestamp_Issues	True if timestamp in the media have been detected,	boolean
File_path	URL or relative path to the media files, respectively for externally hosted files,	string
Favourite	True if it contains videos tagged as favorite,	boolean
Comments	Comments or notes about the revision,	string

Table S.1.3 ***Observations***

Table with video files results from visualization. Associated with deployments (deploymentID) and with revisions (revision_ID) through Videos.file_path. Note that this data will be generated directly with Timelapse software as explained below.

**Name**	**Definition**	**Type**
File	Name of the video file. If more than one video files use concatenate separated by “,”,	string
Path	URL or Relative path to the first Obs.File, respectively for externally hosted files,	string
Plant_sp	Name of the focal plant species,	string
Plant_ID	Unique identifier of the plant individual. Example: Sasp003,	string
DateTime	Date and time at which the video started. Formatted as an ISO 8601 string with timezone designator, (YYYY-MM- DDThh:mm:ss±hh:mm)	datetime
Sp1	Latin binomial for the principal animal species recorded in the video. Example: *Athene noctua*,	string
Behaviour	Primary activity performed by the focal animal (*Sp1*) during the video. Example: *feeding,*	string
Sp2	Latin binomial for a secondary animal species recorded in the video.	string
Behaviour_Sp2	Primary activity performed by the focal animal (*Sp2*) during the video. Example: *agonistic encounter*.	string

Sp3	Latin binomial for a secondary animal species recorded in the video.	string
Behaviour_Sp3	Primary activity performed by the focal animal (*Sp3*) during the video. Example: *searching for food*.	string
N_cam	Number of cameras set in the same focus individual.	number
Videos	Number of videos recorded in the current revision.	number
Days	Number of days for the camera recording in the current revision. Sampling effort for a video in a given revision.	number
Video_events	When videos are collapsed in time (i.e., 5 min) number of videos that where collapsed. It will be coincident with the number of different Obs.File names.	number
Duration	Number of second for the duration of the event. Could be the sum of durations from different Obs.video_events.	number
TimeStamp_Issue	True if timestamp in the video have been detected.	number
Long	Longitude of the deployment location in decimal degrees, using the WGS84 datum.	number
Lat	Latitude of the deployment location in decimal degrees, using the WGS84 datum.	number

S2. Recommendations for fieldwork

In this section, we provide summarized recommendations for conducting effective camera-trap field surveys and optimizing camera settings. As stated in the main paper, behavioral observations require the use of video mode. To optimize memory cards/batteries and post-processing workflow, it is recommended to set the video length consistently to 10-20 s.

The motion trigger should be set with a delay of 1 s or less if possible, to maximize the recording of animal behavior in the scene. However, it is important to note that the motion sensor of some cameras may have a wider angle of sensitivity than the camera lens, which may result in the beginning of the video being empty if the animal is not yet within view. The sensitivity of the camera should be managed with caution, as it varies depending on the camera model/make. To ensure optimal detection, preliminary trials to set sensitivity correctly are strongly recommended. The placement of the camera is critical to achieving effective detection, and it is recommended to place the camera at shoulder height of the target species (Palencia et al. 2021). In practice, for monitoring plant-animal interactions this may not always be possible due to the diverse range of species, including birds and mammals of different sizes and behaviors, that may be present, especially in tropical areas. In such cases, multiple cameras may be necessary to capture the full range of visitors (e.g., covering canopy- and ground-foraging animals). To avoid duplication of data, it is extremely important to ensure that the field-of-view (FOV) of cameras used simultaneously on the same plant target do not overlap.

To optimize field workload and avoid bias in the final dataset, it is recommended to use a single camera model/make when possible. If a set of different makes is used, a systematic rotation of the deployment/camera may be a solution, but this will make the rest of the workflow more challenging. All cameras should be clearly identified with a unique identifier, such as the camera serial number or a 2-digit code for simplicity, and should be set with the same parameters.

Table S.2.1. Recommendations for camera trap settings. Note that these settings correspond to Browning Dark Ops® Trail cams.

Parameter	Recommendation
Date/Time	Set correct date and time; check for timestamp issues in each camera/revision.<br><br>Use **UTC time.
Capture Mode	Video.
Capture Delay	1 second.
Cap start/end	24 hours - if less, correct sampling effort in the database.
Video Quality	Ultra (1600 × 900 @ 24 fps).
Video Length	10 seconds.
Smart IR	OFF – video clip should not continue recording even if motion continues.
Info Strip	ON – mandatory to assist with reviewing timestamp issues.

SD Management	OFF – erase of older pictures is not allowed.
Motion Detect	Power Safe.

S3. Key Considerations and Recommendations for Field Data Collection

By adhering to the following considerations and best practices, the monitoring of plant–animal interactions in Mediterranean ecosystems and beyond can be conducted with improved consistency, accuracy, and overall efficiency.

1. *Focal species/individual selection and camera placement*
   It is important to recognize that not all species are equally suitable for camera trap monitoring. Variations in growth habit, fruiting display, and microhabitat conditions can limit the ability to gather data efficiently. Preference should be given to species that allow for an unobstructed field of view and reliable monitoring of fruit availability and animal interactions. Once target species are selected, ideally covering the range of fleshy-fruiting plants in the study area, a phenology timeline should be developed to guide the timing of camera deployment. Field searches should then focus on identifying individuals bearing ripe fruit. These individuals do not need to be selected at random, as doing so may include plants with low fruit production, poor visibility, or obstructed access, all of which hinder effective monitoring. Instead, individuals should be chosen to reflect a diversity of growing conditions while ensuring suitability for camera placement to maximize interaction records. Ideally, stratified random sampling of individual plants ensures adequate representation of the range of growing site conditions in heterogeneous habitats (e.g., including individuals from forest edge and forest interior in a stratified random sample). Throughout the monitoring period, plant selection should remain dynamic, with ongoing assessment of fruiting status and visibility. Cameras should be installed minimally altering the plant or its surroundings to preserve natural interaction dynamics and avoid affecting foraging preferences of frugivorous animals. Placement should aim to frame the entire plant or the fruit-bearing section, depending on the species' architecture (e.g., full coverage for *Juniperus* spp., partial for *Rubus ulmifolius*) (see examples and additional discussion in Villalva et al. 2024). Ideally, deployment distance to the plant should compromise close distances to ensure individual fruits can be later visualized in the footage, with longer distances to ensure ample areas or the whole canopy are included. When a single camera cannot capture both canopy and ground-level activity (e.g., in temperate forest and tropical areas with tall trees; Koike et al. 2012, Franceschi et al. 2024), multiple cameras should be used to document interactions in both zones, including animal foraging on fallen fruits.

2. *Camera Model and Make*
   To minimize detection bias and maintain data consistency across the study, it is recommended to use a single camera trap model throughout the entire field campaign. Different models may have varying detection capabilities, which can affect data comparability (Palencia et al., 2021).

3. *Sensor Sensitivity Settings*
   Adjust the camera's sensitivity according to environmental conditions and the characteristics of the target plant species.
   - ➔ In open areas with low background movement, high sensitivity can improve detection.
   - ➔ In dense or cluttered environments, lower sensitivity is preferable to reduce false triggers from vegetation or wind.

4. *Camera Height Placement*
   Placing cameras at the shoulder height of the target animal species increases the likelihood of detection. For greater data coverage:
   - ➔ Use multiple cameras at different heights.
   - ➔ When using multiple cameras (e.g. canopy and ground) ensure the fields of view do not overlap, to avoid duplicate recordings.
   - ➔ Ensure adequate support and stable placement to avoid undesired camera displacements during operation.
   - ➔ Avoid altering the surroundings of the area sampled by the camera; avoid cutting or stripping branches, etc., so the foraging behavior of animals is unaltered. For example, some animals use branches and sticks for perching located close to the infructescences; removing them would alter foraging preferences.

5. *Trigger Speed*
   Set the trigger speed to the shortest possible delay, ideally 1 s or less. A faster trigger speed increases the chances of capturing complete animal behaviors as they occur near the focal plant.

6. *Camera Distance from Focal Plant*
   Position the cameras between the model's minimum focus distance and a few meters beyond the focal plant. This distance helps:
   - ➔ Minimize the number of files triggered by wind.
   - ➔ Improve the identification of small animals.
   - ➔ Try to visualize individual fruits in the footage so that fruit foraging details can be recorded, if present.
   - ➔ Ensure enough area of the fruiting canopy is visualized. This usually represents a compromise between closeness to comply with the previous indications and assuring enough distance to visualize as much canopy area of the focal plant as possible.

7. *Sampling Effort Documentation*
   To accurately track sampling effort, record a video at the beginning and end of each camera revision session. This practice ensures a reliable estimation of monitoring duration and supports the integrity of the final dataset by preventing sampling gaps or bias.

Figure S.3. Heatmap of confusion matrices. Heatmap showing normalized confusion matrix components: true positives (TP), true negatives (TN), false positives (FP), and false negatives (FN) for each plant species analyzed. Color intensity represents the proportion of each classification outcome within species, with darker shades indicating higher values. This visualization highlights interspecific variation in computer vision model performance across the different species monitored complementary to Figure 3 in the main text document.

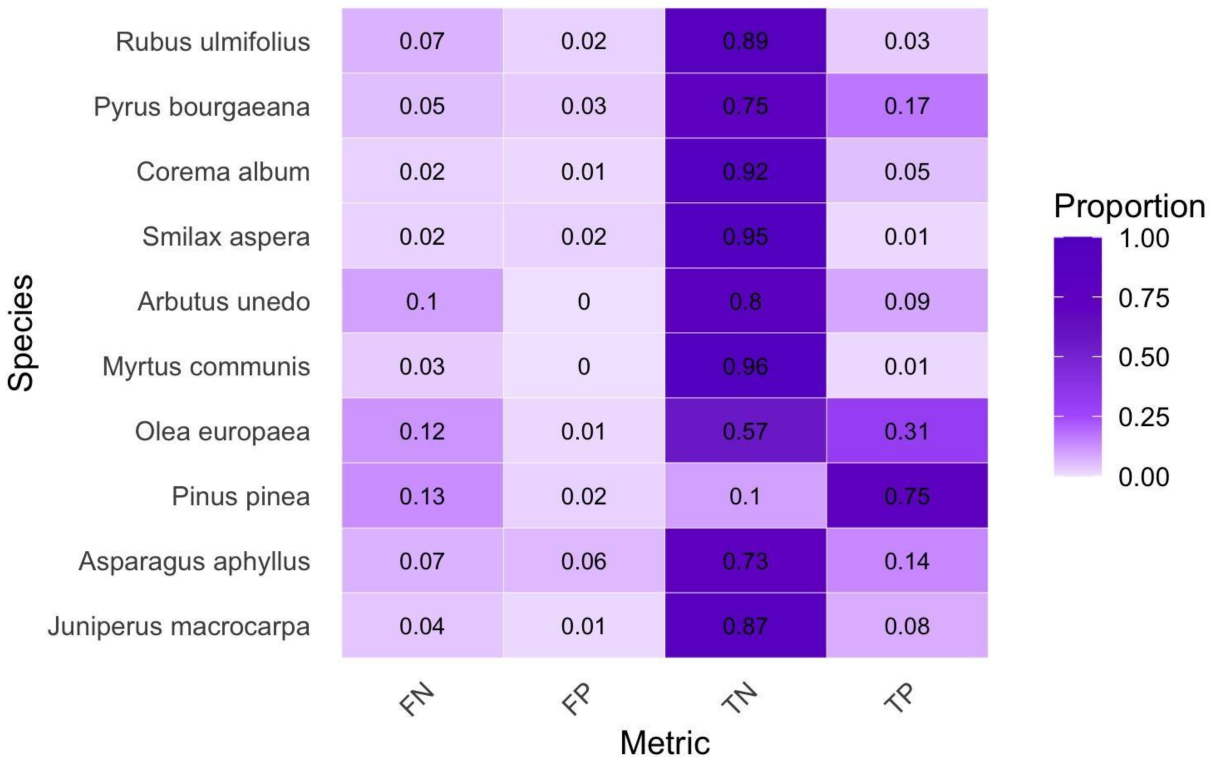

References

Bubnicki, J. W., B. Norton, S. J. Baskauf, T. Bruce, F. Cagnacci, J. Casaer, M. Churski, J. P. G. M. Cromsigt, S. D. Farra, C. Fiderer, T. D. Forrester, H. Hendry, M. Heurich, T. R. Hofmeester, P. A. Jansen, R. Kays, D. P. J. Kuijper, Y. Liefting, J. D. C. Linnell, M. S. Luskin, C. Mann, T. Milotic, P. Newman, J. Niedballa, D. Oldoni, F. Ossi, T. Robertson, F. Rovero, M. Rowcliffe, L. Seidenari, I. Stachowicz, D. Stowell, M. W. Tobler, J. Wieczorek, F. Zimmermann, and P. Desmet. 2024. Camtrap DP: an open standard for the FAIR exchange and archiving of camera trap data. Remote Sensing in Ecology and Conservation 10: 283–295. doi: 10.1002/rse2.374.

Desmet, P., J. Bubnicki, and B. Norton. 2021. Camtrap DP: A frictionless data exchange format for camera trapping data. Biodiversity Information Science and Standards 5: e73188. doi: 10.3897/biss.5.73188.

Franceschi, I. C., R. A. da P. Dornas, I. S. Lermen, …, and I. P. Coelho. 2024. Camera trap surveys of Atlantic Forest mammals: A data set for analyses considering imperfect detection (2004–2020). Ecology 105: e4298. doi: 10.1002/ecy.4298.

Koike, S., H. Morimoto, S. Kasai, Y. Goto, C. Kozakai, I. Arimoto, and K. Yamazaki. 2012. Relationships between the fruiting phenology of *Prunus jamasakura* and timing of visits by mammals - estimation of the feeding period using camera traps. Pp.: 53-68 in X. Zhang, editor. *Phenology and Climate Change*. InTech, Rijeka, Croatia.

Palencia, P., Vicente, J., Soriguer, R. C., & Acevedo, P. (2021). Towards a best-practices guide for camera trapping: Assessing differences among camera trap models and settings under field conditions. *Journal of Zoology*, *316*(3), 197–208.

Villalva, P., B. Arroyo‑Correa, G. Calvo, P. Homet, J. Isla, I. Mendoza, E. Moracho, E. Quintero, F. Rodríguez‑Sánchez, and P. Jordano. 2024. FRUGIVORY CAMTRAP: A dataset of plant–animal interactions recorded with camera traps. Ecology 105: e4424. doi: 10.1002/ecy.4424.

Supplementary S4. Code

The code functions provided in our streamline protocol can be accessed through https://github.com/PJordano-Lab/Ecological-interactions-camtrap-protocol/releases/tag/v1.0.0 and include five main tasks summarized below. Also available at: https://zenodo.org/records/17353294

- ***Video splitting*** - Automates the process of recursive extraction of frames from video files. It organizes the frames into new directories following the same structure as the original ones, and used for running the image detection model at frame level.
- ***Working with json*** - Processes JSON data, filters and selects specific video files based on their confidence levels, moves the selected video files to new directories with modified paths, and combines data from multiple JSON files into a single data frame as input for the visualization software.
- ***Video collapsing*** - Collapses video events in fixed intervals from the 'DateTime' values, and performs various aggregations and calculations for each group.
- ***Obtaining sampling effort*** - Processes video files from parent directories, calculates the duration and number of files for each subdirectory, organizes the results into a data frame, manipulates the data frame to extract relevant information, and writes the processed data to a .csv file for further merging with the video-level dataset.
- ***Obtaining file metadata*** - Reads and processes video files' metadata (e.g., video duration and creation time) from the parent directory, to merge the resulting metadata to the video-level dataset (e.g., EXIF tools).
